# Supplementary material for: Palovarotene reduces heterotopic ossification in juvenile FOP mice but exhibits pronounced skeletal toxicity
Source: eLife. 2018 Sep 18;7:e40814. doi: 10.7554/eLife.40814 (PMC6143342; doi:10.7554/eLife.40814)
Supplement: Figure 6—source data 1. [file elife-40814-fig6-data1.docx]

**Figure 6 - Source Data 1. Two-way ANOVA analysis of bioluminescent FAP population dynamics**

| Day 1 | Significance | Adjusted P Value |
| --- | --- | --- |
| R206H-FAPs vs. R206H-FAPs + PVO | ns^1^ | 0.9022 |
| R206H-FAPs vs. WT FAPs | ** | 0.0079 |
| R206H-FAPs vs. R206H-FAPs + ActA-mAb | ns | 0.1364 |
| R206H-FAPs + PVO vs. WT FAPs | ns | 0.1192 |
| R206H-FAPs + PVO vs. R206H-FAPs + ActA-mAb | ns | 0.5568 |
| WT FAPs vs. R206H-FAPs + ActA-mAb | ns | 0.9604 |
| Day 3 | **Significance** | **Adjusted P Value** |
| R206H-FAPs vs. R206H FAPs + PVO | **** | <0.0001 |
| R206H-FAPs vs. WT FAPs | **** | <0.0001 |
| R206H-FAPs vs. R206H FAPs + ActA-mAb | **** | <0.0001 |
| R206H-FAPs + PVO vs. WT FAPs | **** | <0.0001 |
| R206H-FAPs + PVO vs. R206H-FAPs + ActA-mAb | *** | 0.0008 |
| WT FAPs vs. R206H FAPs + ActA-mAb | ns | 0.5628 |
| Day 5 | **Significance** | **Adjusted P Value** |
| R206H-FAPs vs. R206H-FAPs + PVO | **** | <0.0001 |
| R206H-FAPs vs. WT FAPs | **** | <0.0001 |
| R206H-FAPs vs. R206H-FAPs + ActA-mAb | **** | <0.0001 |
| R206H-FAPs + PVO vs. WT FAPs | **** | <0.0001 |
| R206H-FAPs + PVO vs. R206H-FAPs + ActA-mAb | *** | 0.0009 |
| WT FAPs vs. R206H-FAPs + ActA-mAb | ns | 0.6836 |
| Day 7 | **Significance** | **Adjusted P Value** |
| R206H-FAPs vs. R206H-FAPs + PVO | **** | <0.0001 |
| R206H-FAPs vs. WT FAPs | **** | <0.0001 |
| R206H-FAPs vs. R206H-FAPs + ActA-mAb | **** | <0.0001 |
| R206H-FAPs + PVO vs. WT FAPs | **** | <0.0001 |
| R206H-FAPs + PVO vs. R206H-FAPs + ActA-mAb | ** | 0.0087 |
| WT FAPs vs. R206H-FAPs + ActA-mAb | ns | 0.765 |

^1^ ns: not significant
